# Minimum heart rate and mortality after cardiac surgery: retrospective analysis of the Multi-parameter Intelligent Monitoring in Intensive Care (MIMIC-III) database

**DOI:** 10.1038/s41598-023-29703-9

**Published:** 2023-02-14

**Authors:** Chaodi Luo, Zhenzhen Duan, Ziheng Xia, Qian Li, Boxiang Wang, Tingting Zheng, Danni Wang, Dan Han

**Affiliations:** 1 Department of Cardiology, First Affiliated Hospital of Xi’an Jiaotong University, Yanta West Road 277, Xi’an, 710061 China; 2 Department of Perivascular Surgery, Honghui Hospital of Xi’an Jiaotong University, Youyi East Road 555, Xi’an, 710054 China; 3 School of Electronic Engineering, Xidian University, Taibai South Road 2, Xi’an, 710071 China; 4 Department of Cardiovascular Surgery, First Affiliated Hospital of Xi’an Jiaotong University, Yanta West Road 277, Xi’an, 710061 Shaanxi China

**Keywords:** Cardiology, Diseases, Risk factors

## Abstract

Low heart rate is a risk factor of mortality in many cardiovascular diseases. However, the relationship of minimum heart rate (MHR) with outcomes after cardiac surgery is still unclear, and the association between optimum MHR and risk of mortality in patients receiving cardiac surgery remains unknown. In this retrospective study using the Multi-parameter Intelligent Monitoring in Intensive Care (MIMIC-III) database, 8243 adult patients who underwent cardiac surgery were included. The association between MHR and the 30-day, 90-day, 180-day, and 1-year mortality of patients undergoing cardiac surgery was analyzed using multivariate Cox proportional hazard analysis. As a continuous variable, MHR was evaluated using restricted cubic regression splines, and appropriate cut-off points were determined. Kaplan–Meier curve was used to further explore the relationship between MHR and prognosis. Subgroup analyses were performed based on age, sex, hypertension, diabetes, and ethnicity. The rates of the 30-day, 90-day, 180-day, and 1-year mortalities of patients in the low MHR group were higher than those in the high MHR group (4.1% vs. 2.9%, P < 0.05; 6.8% vs. 5.3%, P < 0.05; 8.9% vs. 7.0%, P < 0.05, and 10.9% vs. 8.8%, P < 0.05, respectively). Low MHR significantly correlated with the 30-day, 90-day, 180-day, and 1-year mortality after adjusting for confounders. A U-shaped relationship was observed between the 30-day, 90-day, 180-day, and 1-year mortality and MHR, and the mortality was lowest when the MHR was 69 bpm. Kaplan–Meier curve analysis also indicated that low MHR had poor prognosis in patients undergoing cardiac surgery. According to subgroup analyses, the effect of low MHR on post-cardiac surgery survival was restricted to patients who were < 75 years old, male, without hypertension and diabetes, and of White ethnicity. MHR (69 bpm) was associated with better 30-day, 90-day, 180-day, and 1-year survival in patients after cardiac surgery. Therefore, effective HR control strategies are required in this high-risk population.

## Introduction

Cardiac surgery is an invasive and complex procedure, and patients after cardiac surgery are particularly vulnerable, because the perioperative condition is burdened by adverse outcomes^1^. Among the many complications that can occur, the most common involves the electrophysiology of the heart^2^. These most often manifest as significant changes in heart rate (HR), including decreased heart rate variability, sinus bradycardia, sinus tachycardia, and arrhythmia^3^. Atrial fibrillation is the most common arrhythmia after cardiac surgery, with an incidence of 20–50%^4–6^. These complications are all important factors leading to the death of patients after cardiac surgery. Several studies previously developed Euroscore I (EI), II (EII), STS score, and SAPS III to evaluate the prognosis of patients with cardiac surgery. However, the predictions of these scoring systems have been unsatisfactory across different cardiac surgery procedures and in different populations^7–10^. Therefore, it is important to identify a faster and easier measurable parameter for high-risk patients after cardiac surgery. Providing standardized, individualized, and precise treatment to such high-risk patients is essential to improve their prognosis.

HR could be obtained easily and non-invasively without invasive training or procedures. As a key factor in adapting cardiac output to metabolic demands, it determines myocardial oxygen demand and coronary blood flow. Due to its regulation by the autonomic nervous system, the heart rate is susceptible to a wide range of ailments^11^. Many cardiovascular diseases, such as acute ischemic stroke, heart failure, and chronic aortic regurgitation, have been demonstrated to be associated with HR as a risk factor for mortality^12–16^. A retrospective cohort study found that critically ill patients with myocardial infarction (MI) and minimal heart rates (MHR) under 60 bpm had higher mortality within 30 days and 1-year^17^. However, the relationship of MHR with outcomes after cardiac surgery is still unclear, and the association between optimum MHR and the risk of mortality in patients receiving cardiac surgery remains unknown.

Hence, this retrospective cohort study was conducted to determine the relationship between MHR and the risk of mortality of patients after cardiac surgery using the data extracted from the Multi-parameter Intelligent Monitoring in Intensive Care III (MIMIC-III) database.

## Methods

### Study population and data

Version 1.4 of the MIMIC-III database was used for data extraction in this study. The MIMIC-III database is a publicly available data set that contains 53,423 ICU admissions to the Beth Israel Deaconess Medical Center in Boston from 2001 to 2012^18^. It was developed by the Massachusetts Institute of Technology’s (MIT) Computational Physiology Laboratory and is the world’s first large-scale intensive care unit database that is open access, provides high-quality data resources for clinical research, and has a wealth of medical data models that are widely accessible to international researchers according to the data usage agreements. In our study, we included 8243 ICU patients undergoing cardiac surgery who were diagnosed based on the Ninth Revision (ICD-9) diagnosis codes and considered eligible for inclusion.

### MHR definitions and outcomes

The patient's HR was measured, verified, and recorded hourly, and the MHR was defined as the patient's lowest HR within 24 h after cardiac surgery. The patients were divided into two groups according to the level of MHR: the low MHR group (MHR < 60 bpm) and the high MHR group (MHR ≥ 60 bpm). The outcomes of our research were defined as 30-day mortality, 90-day mortality, 180-day mortality, and 1-year mortality of patients with cardiac surgery from the date of admission.

### Data acquisition

Data acquisition was performed using structure query language (SQL) in PostgreSQL (v12.2; PostgreSQL Global Development Group). A significant amount of information was collected about each patient at admission, and the following clinical information was extracted: demographic data (age, gender, and ethnicity); nursing progress notes (weight, height, heartbeat, systolic blood pressure [SBP], diastolic blood pressure [DBP], respiratory rate, oxygen saturation [SpO2], vent duration); laboratory results (glucose, white blood cell [WBC], hemoglobin, platelet, blood urine nitrogen [BUN], creatinine); medical history (hypertension, diabetes, chronic obstructive pulmonary disease [COPD], chronic kidney disease [CKD], smoking, alcohol abuse, continuous renal replacement therapy [CRRT]); type of cardiac surgery (coronary artery bypass grafting [CABG], valve surgery only, aortic surgery only, CABG + aortic surgery, valve + aortic surgery, others); medication records (angiotensin-converting enzyme inhibitors (ACEI), statin, proton pump inhibitors [PPI], insulin, metformin, aspirin, warfarin, clopidogrel); and transfer records (length of stay in ICU, length of stay in hospital), Glasgow coma score [GCS] which was used to determine the severity of illness at ICU admission, simplified acute physiological state score [SAPSIII score], sequential organ failure assessment [SOFA] score, logistic organ dysfunction system [LODS] score, and the Oxford acute severity of illness score [OASIS]).

### Statistical analysis

The number and percentage were presented for categorical data and were compared using the Fisher’s exact test or Pearson’s chi-square test. Continuous variables were checked for normality, and normally distributed variables were reported as the mean ± standard deviation and compared by the Student’s *t* test, while non-normally distributed variables were reported as medians with interquartile ranges (IQRs) and compared by the Kruskal–Wallis test.

Multivariable Cox proportional hazard analysis was used to determine whether MHR was independently associated with the 30-day, 90-day, 180-day, and 1-year mortalities after adjusting for confounders. Model 1, univariate Cox regression analysis of MHR with mortality; Model 2 adjusted for model 1 plus gender, age, and ethnicity; Model 3 adjusted for model 2 plus SBP, DBP, and SOFA; Model 4 adjusted for model 3 plus hypertension, CKD, COPD, and diabetes; and Model 5 adjusted for model 4 plus usage of beta blocker, milrinone, dobutamine, dopamine, and norepinephrine. The log-rank test was used to compare the Kaplan–Meier survival curves between the low and high MHR groups. The MHR was also analyzed as a continuous variable using restricted cubic splines to identify potential non-linear relationships with crude hazard ratios and adjusted hazard ratios. Subgroup analysis were performed to determine the confounding impact of various groups, which was based on types of cardiac surgery, age, hypertension, and ethnicity. All statistical analyses were processed using SPSS software (version 23.0, IBM Corporation, NY, USA) and R programming language (version 4.0.0, R Foundation for Statistical Computing, Vienna, Austria). All P values < 0.05 were considered to indicate statistical significance.

## Results

### Baseline characters

In all, 8351 patients who underwent cardiac surgery were included in the MIMIC-III database, and 8243 patients met the inclusion criteria of our study. Patients classified according to the MHR category have the following percentages: low MHR group (MHR < 60 bpm), 16.7% (n = 1376) and high MHR group (MHR ≥ 60 bpm), 83.3% (n = 6867). The baseline characteristics of patients based on MHR category are presented in Table 1. Patients in the low MHR group (71.73 ± 27.49 years) were older than those in the high MHR group (68.19 ± 27.32 years). Patients in the low MHR group had significantly lower admission HR (72.30 ± 9.64 vs. 86.79 ± 9.44, P < 0.001). The SBP was higher in the low MHR group than in the high MHR group, while the DBP was comparatively lower in the low MHR group (P < 0.001). It was more likely that patients with low MHR would have lower respiratory rate (16.95 ± 2.90 vs. 17.50 ± 3.13, P < 0.001) and SpO_2_ (97.66 ± 2.00 vs. 97.92 ± 1.46, P < 0.001). The low MHR group had significantly higher SAPSIII score [36 (28–47) vs. 34 (27–45), P = 0.002] and GCS score [15 (15–15) vs. 15 (14–15), P = 0.013] than the high MHR group, as well as vent duration and length of ICU stay [2.42 (1.30–4.69) vs. 2.19 (1.23–4.04), P < 0.001], while the SOFA score [4 (2–6) vs. 5 (3–6), P < 0.001]; SIRS score [3 (2–3) vs. 3 (3–4), P < 0.001]; and LODSs score [4 (2–6) vs. 4 (3–6), P = 0.001] in the low MHR group were lower. Meanwhile, the proportions of cardiac surgery such as CABG (43.6% vs. 47.0%, P = 0.023); aortic surgery (1.8% vs. 0.7%, P < 0.001); valve surgery (12.7% vs. 14.1%, P = 0.172); CABG + aortic surgery (0.2% vs. 0.1%, P = 0.135); valve + aortic surgery (19.8% vs. 24.2%, P < 0.001) and other invasive cardiac surgery (21.9% vs. 13.9%, P < 0.001) in the low MHR group were all lower than the high MHR group. Importantly, the 30-day mortality was higher in the low MHR group (4.1% vs. 2.9%), as well as 90-day mortality (6.8% vs. 5.3%), 180-day mortality (8.9% vs. 7.0%), and 1-year mortality (10.9% vs. 8.8%) (P < 0.05).

**Table 1:** Characteristics of participants categorized by MHR.

Characteristics	Low MHR group (< 60 bpm)	High MHR group (≥ 60 bpm)	P
N	1376	6867	–
Age, years	71.73 ± 27.49	68.19 ± 27.32	< 0.001
Weight	82.60 ± 18.91	83.38 ± 24.08	0.266
Male	931 (67.7)	4590 (66.8)	0.572
Ethnicity (white)	1018 (74.0)	4889 (71.2)	0.036
height	170.82 ± 12.84	170.49 ± 13.09	0.414
BMI	28.34 ± 5.86	29.61 ± 4.71	0.556
Heart rate, bpm	72.30 ± 9.64	86.79 ± 9.44	< 0.001
SBP, mmHg	116.08 ± 12.09	112.42 ± 9.98	< 0.001
DBP, mmHg	56.09 ± 8.12	57.13 ± 7.13	< 0.001
Respiratory rate	16.95 ± 2.90	17.50 ± 3.13	< 0.001
SpO_2_	97.66 ± 2.00	97.92 ± 1.46	< 0.001
Glucose	137.91 ± 34.30	137.04 ± 30.76	0.344
WBC	12.57 ± 5.26	12.51 ± 4.98	0.682
Hemoglobin	10.30 ± 1.70	10.24 ± 1.46	0.525
Platelet	193.38 ± 92.47	193.60 ± 88.59	0.935
BUN	20.26 ± 14.08	20.29 ± 13.90	0.934
Creatinine	0.90 (0.70–1.10)	0.90 (0.70–1.15)	0.377
Hypertension	151 (11.1)	564 (8.2)	< 0.001
COPD	217 (15.8)	1113 (16.2)	0.689
Diabetes	417 (30.3)	2173 (31.6)	0.340
CKD	166 (12.1)	671 (9.8)	0.011
Alcohol abuse	34 (2.5)	177 (2.6)	0.852
Smoking	973 (70.86)	4871 (70.95)	0.246
CRRT	55 (4.0)	302 (4.4)	0.505
GCS	15 (15–15)	15 (14–15)	0.013
SOFA	4 (2–6)	5 (3–6)	< 0.001
SIRS	3 (2–3)	3 (3–4)	< 0.001
SAPSIII	36 (28–47)	34 (27–45)	0.002
LODS	4 (2–6)	4 (3–6)	0.001
OASIS	31 (26–36)	31 (27–36)	< 0.001
Vent duration	9 (4.33–20.47)	7 (3.9–18.5)	< 0.001
Los ICU	2.42 (1.30–4.69)	2.19 (1.23–4.04)	< 0.001
Los hospital	8.17 (5.67–12.98)	7.97 (5.40–12.51)	0.192
Surgery type			
CABG	600 (43.6)	3225 (47.0)	0.023
Valve surgery	175 (12.7)	971 (14.1)	0.172
Aortic surgery	25 (1.8)	46 (0.7)	< 0.001
CABG + Aortic	3 (0.2)	5 (0.1)	0.135
Valve + Aortic	272 (19.8)	1663 (24.2)	< 0.001
Other	301 (21.9)	957 (13.9)	< 0.001
Drugs			
ACEI	345 (25.1)	1226 (17.9)	< 0.001
Statin	827 (60.1)	4050 (59.0)	0.453
PPI	724 (52.6)	3384 (49.3)	0.025
Insulin	1135 (82.5)	5734 (83.5)	0.362
Metformin	116 (8.4)	706 (10.3)	0.038
Aspirin	1070 (77.8)	5563 (81.0)	0.006
Clopidogrel	193 (14.0)	942 (13.7)	0.764
Warfarin	317 (23.0)	1619 (23.6)	0.676
Beta blocker	1154 (83.9)	5846 (85.1)	0.232
Milrinone	110 (8.0)	855 (12.5)	< 0.001
Dobutamine	30 (2.2)	224 (3.3)	0.033
Dopamine	98 (7.1)	199 (2.9)	< 0.001
Norepinephrine	144 (10.5)	922 (13.4)	0.003
ICU mortality	43 (3.1)	149 (2.2)	0.039
In-hospital mortality	50 (3.6)	192 (2.8)	0.096
30-day mortality	56 (4.1)	196 (2.9)	0.020
90-day mortality	93 (6.8)	361 (5.3)	0.028
180-day mortality	123 (8.9)	480 (7.0)	0.013
1-year mortality	150 (10.9)	604 (8.8)	0.014

Participants were divided into two groups, a low MHR group (MHR < 60 bpm) and a high MHR group (MHR ≥ 60 bpm). For each variable, mean ± standard deviation, median (interquartile range), or number (percent) was reported (as appropriate).

*BMI* body mass index, *SBP* systolic blood pressure, *DBP* diastolic blood pressure, *WBC* white blood cell, *BUN* blood urea nitrogen, *COPD* chronic obstructive pulmonary disease, *CKD* chronic kidney disease, *CRRT* continuous renal replacement therapy, *GCS* glasgow coma score, *SOFA* sequential organ failure assessment, *SIRS* systemic inflammatory response syndrome, *SAPS* simplified acute physiological state score, *LODS* logistic organ dysfunction system, *OASIS* oxford acute severity of illness score, *CABG* coronary artery bypass grafting, *ACEI* angiotensin-converting enzyme inhibitors, *PPI* proton pump inhibitors.

### Relationship between MHR and the clinical outcomes of patients after cardiac surgery

We analyzed the relationship between MHR and clinical outcomes after cardiac surgery using Cox proportional hazards regression model. Model 1 indicated greater risks for 30-day, 90-day, 180-day, and 1-year for the low MHR group (all P < 0.05) than the high MHR group. Similarly, model 4 adjusted for age, gender, ethnicity, SBP, DBP, SOFA, and several comorbidities such as hypertension, CKD, COPD, and diabetes, showed that people with low MHR were at greater risk for mortality than those with high MHR. Model 5 was further adjusted for beta blocker, milrinone, dobutamine, dopamine, and norepinephrine usage, and low MHR remained significantly associated with 30-day, 90-day, 180-day, and 1-year mortality with hazard ratios of 1.594 [95% confidence interval CI 1.178–2.157], 1.351 (95% CI 1.071–1.705), 1.334 (95% CI 1.090–1.633), and 1.286 (95% CI 1.072–1.544), respectively (Table 2).

**Table 2:** Association between MHR group and the outcomes of patients after cardiac surgery.

Model	30-day mortality		90-day mortality		180-day mortality		1-year mortality	
	Hazard ratio (95% CI)	P	Hazard ratio (95% CI)	P	Hazard ratio (95% CI)	P	Hazard ratio (95% CI)	P
Model 1	1.429 (1.062–1.923)	0.019	1.293 (1.030–1.624)	0.027	1.290 (1.058–1.573)	0.012	1.254 (1.049–1.500)	0.013
Model 2	1.417 (1.053–1.908)	0.021	1.276 (1.016–1.603)	0.036	1.274 (1.045–1.554)	0.017	1.240 (1.037–1.483)	0.018
Model 3	1.585 (1.174–2.139)	0.003	1.382 (1.096–1.742)	0.006	1.396 (1.143–1.706)	0.001	1.320 (1.103–1.580)	0.002
Model 4	1.571 (1.163–2.121)	0.003	1.387 (1.101–1.746)	0.005	1.358 (1.112–1.660)	0.003	1.282 (1.070–1.534)	0.007
Model 5	1.594 (1.178–2.157)	0.003	1.351 (1.071–1.705)	0.011	1.334 (1.090–1.633)	0.005	1.286 (1.072–1.544)	0.007

Hazard ratio and 95% CI for MHR group in 30-day, 90-day, 180-day and 1-year mortality were calculated using different Cox regression models. Model 1, univariate Cox regression analysis of MHR with mortality; Model 2 adjusted for model 1 plus gender, age, ethnicity; Model 3 adjusted for model 2 plus SBP, DBP,SOFA; Model 4 adjusted for model 3 plus hypertension, CKD, COPD, diabetes; Model 5 adjusted for model 4 plus beta blocker, milrinone, dobutamine, dopamine, norepinephrine.

### Study outcomes

**Figure 1:**
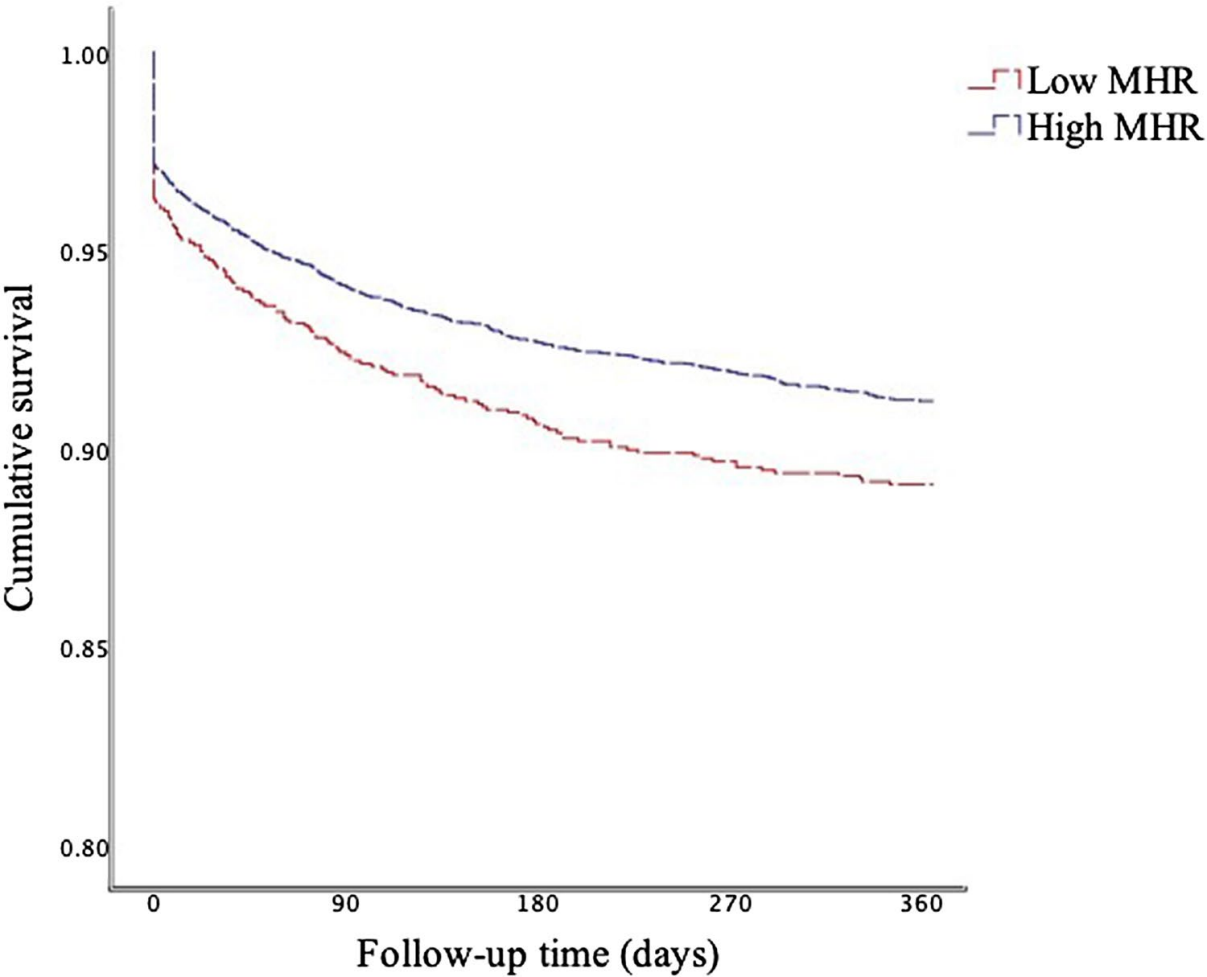
Kaplan–Meier curves of 1-year mortality by MHR.

Clinical outcomes were measured by Kaplan–Meier curve in our study. Figure 1 shows the Kaplan–Meier survival curve of 1-year mortality, indicating that the low MHR group has a significant disadvantage over the high MHR group in terms of 1-year survival (log-rank test P < 0.05).

### “U-type” association between MHR and outcomes

**Figure 2:**
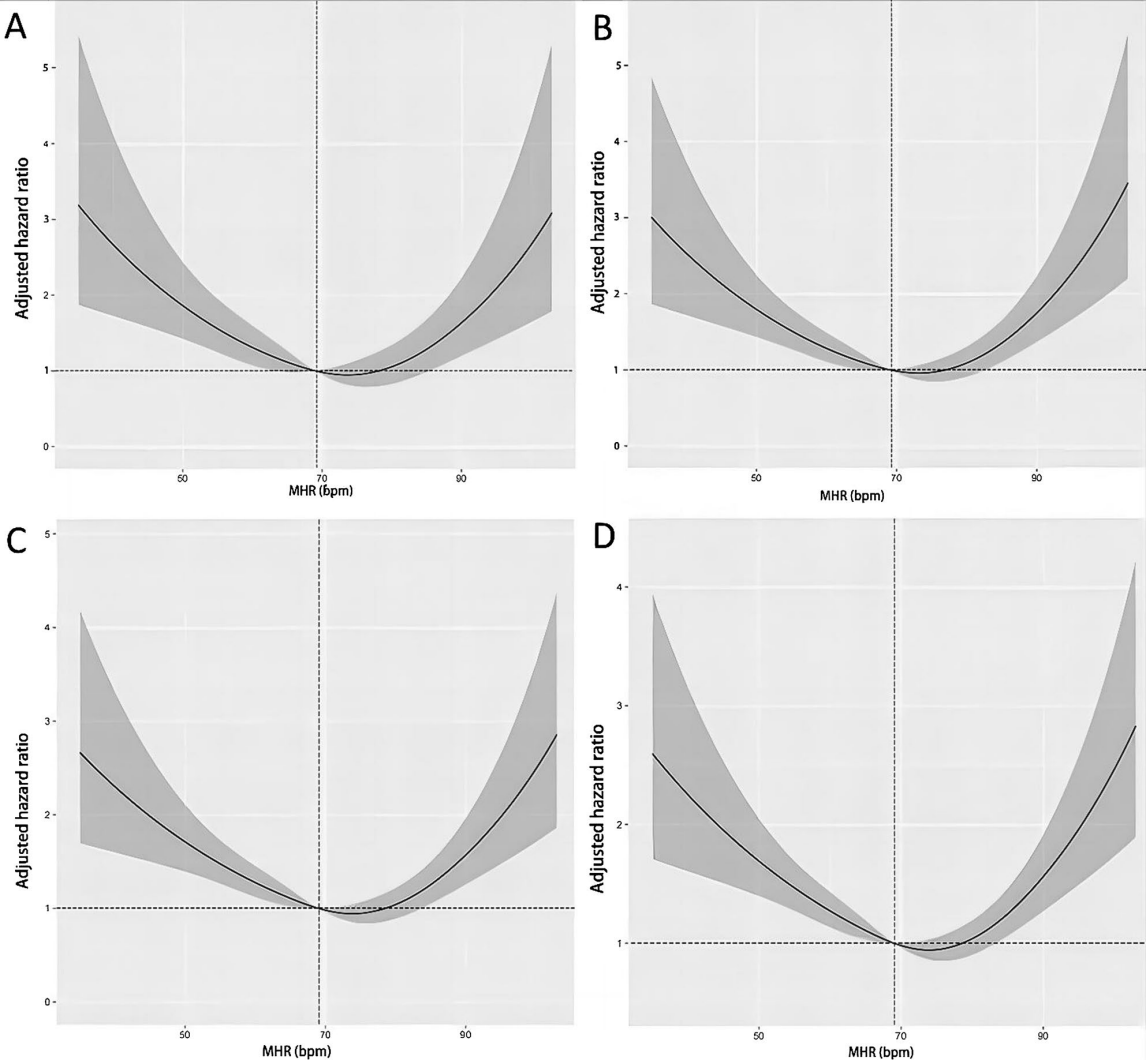
Association between MHR and outcomes of patients undergoing cardiac surgery. Adjusted hazard ratio and 95% CI for MHR in 30-day mortality (**A**), 90-day mortality (**B**), 180-day mortality (**C**), and 1-year mortality (**D**). Analyses were conducted using a model based on RCS. The reference (hazard ratio = 1, horizontal dotted line) was an MHR of 69 bpm (vertical dotted line). Adjusted variables included age, gender, ethnicity, SOFA score, SBP, DBP, hypertension, diabetes, CKD, and COPD. *MHR* minimum heart rate, *SOFA* sequential organ failure assessment, *SBP* systolic blood pressure, *DBP* diastolic blood pressure, *CKD* chronic kidney disease, *COPD* chronic obstructive pulmonary disease.

We also examined MHR as a continuous variable and determined a cut-off of 69 bpm using restricted cubic splines (RCS). MHR and outcomes of patients with cardiac surgery in the ICU were found to have an apparent nonlinear relationship when we used RCS analysis. The correlation between MHR and 30-day (Supplementary Fig. 1A), 90-day (Supplementary Fig. 1B), 180-day (Supplementary Fig. 1C), and 1-year (Supplementary Fig. 1D) outcomes could be characterized as a “*U*-type” curve. Further adjusting for a series of covariates, the relationship between MHR and 30-day (Fig. 2A), 90-day (Fig. 2B), 180-day (Fig. 2C), and 1-year (Fig. 2D) mortality could also be characterized as a “*U*-type” curve. The mortality was lowest when MHR was 69 bpm. The results of the RCS model showed that the risk of death decreased with the increase of discharge time. Patients with cardiac surgery had a higher 30-day mortality and 90-day mortality than 180-day mortality, and the 1-year mortality was the lowest, regardless of the adjustment for covariates.

### Subgroup analysis

Based on age levels, gender, hypertension, diabetes, and ethnicity, subgroup analyses were conducted (Table 3). Of the surgery type subgroups, compared to the high MHR group, the results of the relationship between types of surgery and 30-day, 90-day, 180-day, and 1-year mortality were not significant. The hazard ratios were still significant in subgroups of age < 75 years, male sex, and White ethnicity, as well as in patients without diabetes and hypertension, while there was no statistical significance in patients with diabetes and hypertension. The correlation between MHR and the 90-day, 180-day, and 1-year mortality were statistically significant (all P < 0.05) in patients with non-hypertension. Whereas, there was no difference in hypertensive patients. Furthermore, the outcome risk of low MHR varied among ethnic groups, and the correlations between low MHR and 30-day, 180-day, and 1-year outcomes were all statistically significant (all P < 0.05) in White patients.

**Table 3:** Association between MHR group and 30-day, 90-day, 180-day and 1-year mortality of patients with cardiac surgery in different subgroups.

Subgroup		N	Hazard ratio (95% CI)							
			30-day	P	90-day	P	180-day	P	1-year	P
Age	< 75	5892	1.826 (1.242–2.685)	0.002	1.571 (1.157–2.134)	0.004	1.498 (1.133–1.980)	0.005	1.338 (1.037–1.725)	0.025
	≥ 75	2351	1.102 (0.691–1.759)	0.683	1.133 (0.805–1.594)	0.474	1.080 (0.815–1.431)	0.591	1.021 (0.793–1.313)	0.874
Gender	Male	5521	1.449 (1.119–2.124)	0.047	1.403 (1.056–1.864)	0.020	1.316 (1.107–1.704)	0.037	1.262 (0.997–1.597)	0.053
	Female	2722	1.407 (0.878–2.256)	0.156	1.133 (0.772–1.661)	0.524	1.264 (0.928–1.722)	0.138	1.255 (0.953–1.652)	0.106
Hypertension	No	7528	1.391 (0.997–1.941)	0.052	1.306 (1.009–1.692)	0.043	1.290 (1.030–1.614)	0.027	1.257 (1.027–1.538)	0.026
	Yes	715	1.360 (0.702–2.633)	0.362	1.036 (0.639–1.680)	0.885	1.073 (0.705–1.633)	0.743	1.034 (0.704–1.520)	0.864
Diabetes	No	5653	1.457 (1.028–2.065)	0.034	1.342 (1.024–1.758)	0.033	1.348 (1.064–1.709)	0.014	1.318 (1.064–1.632)	0.012
	Yes	2590	1.350 (0.765–2.382)	0.300	1.182 (0.773–1.810)	0.440	1.172 (0.817–1.683)	0.338	1.128 (0.814–1.562)	0.469
Ethnicity	White	5907	1.515 (1.062–2.161)	0.022	1.485 (1.006–2.194)	0.047	1.305 (1.028–1.656)	0.028	1.243 (1.002–1.542)	0.047
	Other	2336	1.286 (0.747–2.216)	0.364	1.222 (0.923–1.618)	0.162	1.286 (0.899–1.838)	0.168	1.305 (0.947–1.798)	0.104

Hazard ratios of 30-day, 90-day, 180-day and 1-year mortality risk on the stratification of age levels, gender, hypertension, diabetes and ethnicity. Patients in high MHR group acts as the reference group. Adjusted variables included gender, age, ethnicity, SBP, DBP, SOFA, hypertension, CKD, COPD, diabetes.

*CI* confidence interval, *SOFA* sequential organ failure assessment, *SBP* systolic blood pressure, *DBP* diastolic blood pressure, *CKD* chronic kidney disease, *COPD* chronic obstructive pulmonary disease.

## Discussion

In this retrospective cohort study, we analyzed 8243 patients who underwent cardiac surgery and divided them into the high and low MHR groups a according to the cut-off point of 60 bpm. We observed that the low MHR group had higher risk for 30-day, 90-day, 180-day, and 1-year mortality than the high MHR group. Additionally, we found a *U*-shaped relationship between MHR and 30-day, 90-day, 180-day, and 1-year mortality based on the RCS model. Based on these data, MHR may be used to predict critically ill patients who received cardiac surgery with poor prognosis, demonstrating the necessity of HR control after cardiac surgery.

Studies have demonstrated that the HR is a risk factor to predict adverse cardiac events and all-cause mortality in patients with diabetes^19^, and cardiovascular diseases including myocardial infarction (MI)^20^, hypertension^21^, atherosclerosis^22^, plaque rupture^23^, heart failure^24,25^, and even in healthy individuals^26^. In critically ill MI patients, Wang et al.^17^ found that MHR < 60 bpm increased the mortality risk at 30-day and 1-year. They also found an L-shaped relationship between the MHR and mortality, which is different from our finding. Lang et al.^27^ observed that the low admission HR (< 60 bpm) was related to the increased mortality in patients with ST-segment elevation myocardial infarction (STEMI) undergoing percutaneous coronary intervention. In addition, bradycardia appears to be an important early warning sign of impending and unexpected cardiac arrest during routine laparoscopic surgery^28^. However, several studies have shown that bradycardia (< 60 bpm) was not an independent risk factor for mortality in STEMI patients^29,30^. Zheng et al. found that only patients with higher HR (≥ 78 beats/min) were at increased risk of adverse outcomes than those with lower HR, after adjusting for several variables^31^. Our data revealed that patients in the low MHR group (< 60 bpm) showed a higher predictive value of 30-day, 90-day, 180-day, and 1-year mortality than the high MHR group. Since HR is very easy to measure after admission, we explored whether the MHR could be used as a convenient and quick parameter to predict the prognosis of patients undergoing cardiac surgery.

There is a non-linear relationship between HR and adverse outcomes that is being explored in emerging research^29,32,33^. Parodi et al.^28^ found that elevated HR (≥ 80 bpm) identifies higher risk of death in patients with AMI undergoing primary PCI, but it is unknown whether HR reduction will result in improved outcome in these patients. Böhm et al.^32^ observed that resting HR > 75 bpm was associated with higher risk of cardiovascular events in diabetic and non-diabetic patients. Nevertheless, these conclusions still need to be researched and validated in prospective trials. To further investigate the relationship between MHR and prognosis of patients after cardiac surgery, we used the RCS model to explore the MHR with the best prognosis. Our study showed a typical *U*-type curve in the RCS model, indicating that an apparent non-liner relationship existed between MHR and 30-day, 90-day, 180-day, and 1-year mortality, and the lowest mortality was at an MHR of 69 bpm. We found that MHR, a physiological parameter easily collected on the first day of admission, is closely associated with the short- and long-term mortality of cardiac surgery patients. We hope that MHR will serve as a rapid marker for identifying high-risk patients in ICUs who are preparing to undergo cardiac surgery.

HR has been considered a reliable risk factor for cardiovascular disease, and beta-blocker^34^ and ivabradine^32,35^-based HR lowering therapy improves cardiovascular outcomes in patients with elevated HR, but not in patients with low HR. For patients with cardiogenic shock in ICUs, pacemaker optimization can be a viable therapeutic option, while increasing cardiac output and reducing catecholamines^36^. As a result of the present study, it is important to note that it may be beneficial to control the MHR around 69 bpm after cardiac surgery for the purpose of lowering the HR.

Previous report provides strong evidence for the linear decline of HR with age in healthy population^37^. HR is an independent predictor of mortality, which varies by age, sex, and disease^38^. Therefore, age and gender were important adjusting factors in our study. Moreover, HR is likely affected by comorbidities such as arterial fibrillation, hypertension, and diabetes; drugs such as anti-hypertensive agents and vasopressors; and ethnicity. For example, Venkatesan et al. found a significant dose-dependent association between low preoperative BP values and increased postoperative mortality in the elderly^39^. African-American patients did not experience higher rates of complications, but they were at higher odds of mortality after experiencing a complication^40^. In the present study, subgroup analysis in patients with and without hypertension or diabetes, types of operation, and ethnicity indicated that the low MHR group had higher risk in patients without hypertension and white ethnicity, but had no difference in patients with hypertension and diabetes. These results reflect that MHR could act as an early risk factor which is convenient to measure in patients after cardiac surgery. In addition, the predictive value of low MHR varies in different type of populations; therefore, larger sample size and multicenter cohort studies are needed to further explore the effect of MHR on patients undergoing cardiac surgery.

## Limitations

First, selection bias could not be excluded in the retrospective cohort study due to its intrinsic design defect. Our results were supported by sensitivity analysis, and further external validation should be conducted to increase the credibility. Second, it is possible that a few patients may have been missed because they were identified using ICD-9 codes instead of clinical diagnostic criteria.

## Conclusions

The present retrospective cohort study showed that the MHR of 69 bpm was associated with lower 30-day, 90-day, 180-day, and 1-year mortality in patients after cardiac surgery. Therefore, effective HR control strategies are required in this high-risk population.

## Supplementary Information

Supplementary Figure 1.

## Data Availability

Publicly available datasets were analyzed in this study. This data can be found here: https://mimic.mit.edu/docs/gettingstarted/.
