# Supplementary material for: Minimum heart rate and mortality after cardiac surgery: retrospective analysis of the Multi-parameter Intelligent Monitoring in Intensive Care (MIMIC-III) database
Source: Sci Rep. 2023 Feb 14;13:2597. doi: 10.1038/s41598-023-29703-9 (PMC9929057; doi:10.1038/s41598-023-29703-9)
Supplement: Supplementary file 1 — Supplementary Figure 1. [file 41598_2023_29703_MOESM1_ESM.docx]

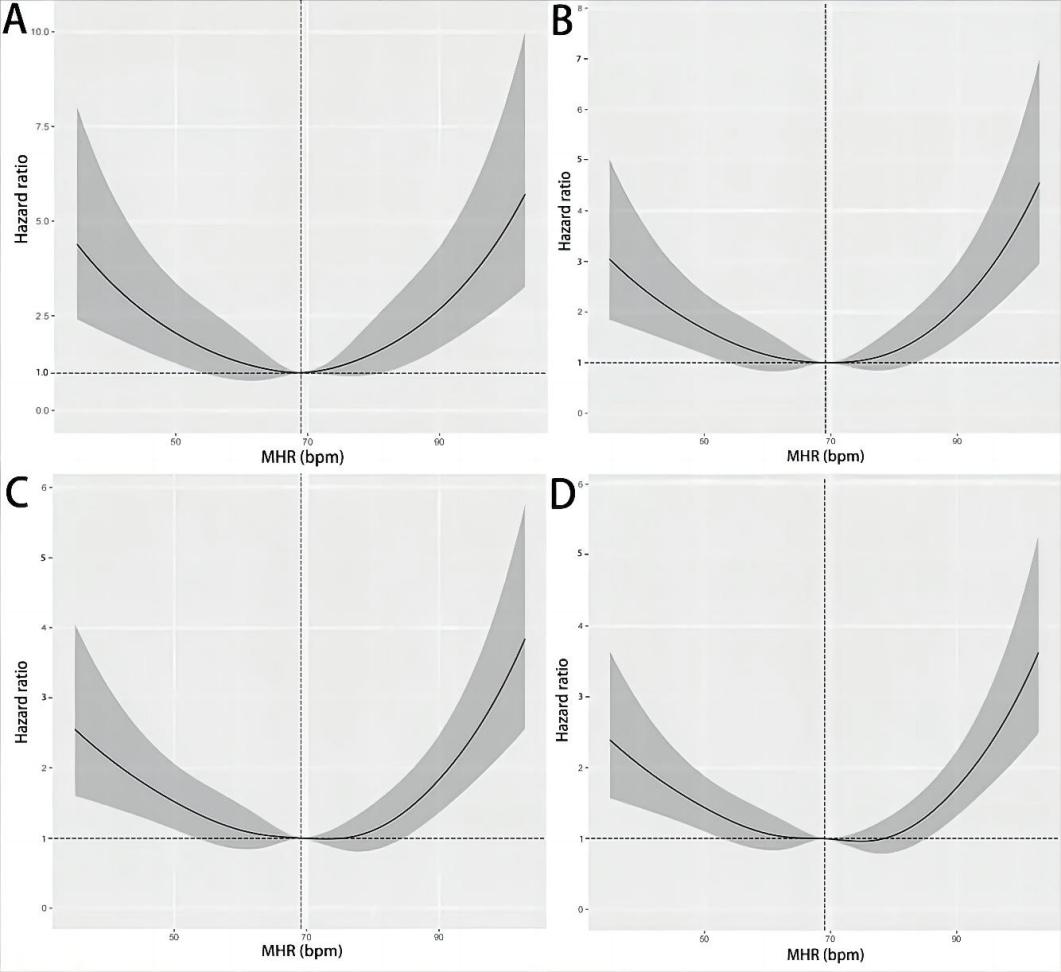


**Suppl Figure 1.** Association between MHR and outcomes of patients undergoing cardiac surgery. Hazard ratio and 95% CI for MHR in 30-day mortality (A), 90-day mortality (B), 180-day mortality (C) and 1-year mortality (D). Analyses were conducted using a model based on RCS. The reference (hazard ratio =1, horizontal dotted line) was an MHR of 69 bpm (vertical dotted line).
